# Co-enhancement of fluorescence and singlet oxygen generation by silica-coated gold nanorods core-shell nanoparticle

**DOI:** 10.1186/1556-276X-9-666

**Published:** 2014-12-10

**Authors:** Xuebin Ke, Dong Wang, Changqing Chen, Anqi Yang, Yu Han, Lei Ren, Donghui Li, Hongjun Wang

**Affiliations:** Department of Biomaterials, College of Materials, Xiamen University, Xiamen, 361005 People’s Republic of China; State Key Laboratory for Physical Chemistry of Solid Surfaces, Department of Chemistry, College of Chemistry and Chemical Engineering, Xiamen University, Xiamen, 361005 People’s Republic of China; Department of Orthopaedic Surgery, The Affiliated Southeast Hospital of Xiamen University, Orthopaedic Center of People’s Liberation Army, Zhangzhou, 363000 People’s Republic of China; College of Medicine, Xiamen University, Xiamen, 361005 People’s Republic of China; Department of Chemistry, Chemical Biology, and Biomedical Engineering, Stevens Institute of Technology, Hoboken, NJ 07030 USA

**Keywords:** Surface plasmon resonance, Silica-coated gold nanorods, Metal-enhanced fluorescence, Metal-enhanced singlet oxygen generation

## Abstract

Metal-enhanced fluorescence (MEF) as a newly recognized technology has been attracting considerable attention and is widely used in fluorescence-based technology. In this paper, we reported a novel distance-dependent MEF and metal-enhanced singlet oxygen generation phenomenon based on silica-coated gold nanorods (AuNRs@SiO_2_) core-shell structure with tetra-substituted carboxyl aluminum phthalocyanine (AlC_4_Pc) that serve as both fluorophore and photosensitizer. When the AlC_4_Pc was linked on the surface of AuNRs@SiO_2_, the fluorescence intensity and singlet oxygen productivity varied with the thickness difference of silica shell from 2.1 to 28.6 nm. The co-enhancement effect reached the maximum of 7-fold and 2.1-fold, respectively, when the separation distance was 10.6 nm. These unique characteristics make the prepared core-shell nanoparticles promising for MEF-based biological imaging and photodynamics therapy.

## Background

Noble metal nanoparticles (NPs) have been widely applied in chemical and biological sensing [1–4], surface-enhanced Raman scattering (SERS) [5, 6], and bioimaging technologies [7, 8] due to their unique shape- and size-dependent localized surface plasmon resonance (LSPR) and local electromagnetic field features. It has also been found that many kinds of metal NPs can dramatically enhance or quench the fluorescence of fluorophores along with the changing of separation distance between them [9]. Such fluorescence enhancement is known as metal-enhanced fluorescence (MEF). Furthermore, when the wavelength of fluorescence emission overlap well with the plasmonic response of metal NPs, the fluorophores can couple with metal NPs, which result in a shortening of lifetime and an increasing of photostability as compared with that of free fluorophores [10, 11]. Therefore, the metal NP-associated fluorophores have illustrated a promising candidate for cell imaging and tracking owing to this fluorophore-metal interaction [11, 12].

Until now, numerous nanodevices have been used to investigate the mechanism of MEF. Most studies of MEF were based on silver island films (SIFs) with the depositing of Ag NPs on a glass substrate [12–14]. However, these structures exhibited irregular morphology and unsuitable size, which would limit their *in vivo* application research. In some other distance-dependent MEF studies, flexible molecules like DNA [15, 16], polyethylene glycol [17], and some other polymers [18, 19] were used as spacer layers, while they were not rigid enough to obtain accurate distance control. To overcome these problems, Gerritsen et al. [20] have developed an AgNPs@SiO_2_ core-shell structure to achieve well performance in MEF by taking the advantage of the easy and accurate tunable feature of silica shell. Such method introduced a new way to manage the distance between fluorophores and metal core in the study of MEF. In addition to MEF, Geddes et al. [21] have found that the photon-induce excited states could enhance both fluorescence and phosphorescence emission, like the phosphorescence emission of singlet oxygen (^1^O_2_) at 1,270 nm. The production of reactive oxygen species (ROS) could be enhanced by the intensified electromagnetic field when the photosensitizers (PSs) were placed in close proximity to the metal NP.

To achieve the co-enhancement of fluorescence intensity and ^1^O_2_ generation, an ideal model system of silica-coated gold nanorod (AuNRs@SiO_2_) with tetra-substituted carboxyl aluminum phthalocyanine (AlC_4_Pc) linked on the surface to serve as both fluorophore and photosensitizer was demonstrated in our work. Gold nanorods were chosen as MEF substrates, which offered the possibility of plasmon resonance tuning to match the spectra of AlC_4_Pc. The distance between AuNRs core and AlC_4_Pc was controlled by varying the thickness of silica shell spacer layers. By adjusting this spacer layer to an appropriate distance, both of the fluorescence intensity and ^1^O_2_ generation of AlC_4_Pc was obviously enhanced by the AuNRs@SiO_2_ core-shell structure. Our current results shed a new light on the PSs delivery system for the real-time visualization of photodynamics therapy (PDT) by using metal-enhanced effect.

## Methods

### Materials

Chloroauric acid (HAuCl_4_ · 3H_2_O), silver nitrate (AgNO_3_), sodium borohydride (NaBH_4_), cetyltrimethyl ammonium bromide (CTAB), L-ascorbic acid (AA), tetraethyoxysilane (TEOS), and dimethyl sulfoxide (DMSO) were purchased from Sinopharm Chemical Reagent Co., Ltd (Sinopharm Chemical Reagent Co., Ltd, Shanghai, China). 3-aminopropyl triethoxysilane (APTMS), 9, 10-anthracenediyl-bis (methylene) dimalonic acid (ABDA), 1-(3-dimethylaminopropyl)-3-ethylcarbodiimide hydrochloride (EDC · HCl), and N-hydroxysuccinimide (NHS) were obtained from Sigma-Aldrich (Sigma-Aldrich St Louis, MO, USA). AlC_4_Pc was synthesized and purified according to a method in the literature [22]. The other reagents were analytical grade and used without further purification. Milli-Q water (less than 18.2 MΩ) was used throughout the synthesis experiments.

### Synthesis of AuNRs

AuNRs were prepared by the well-known seed-mediated growth process [23]. The seed solution was prepared firstly; 9.5 mL of 0.1 M CTAB solution was mixed with 0.25 mL of 0.01 M HAuCl_4_. After that, a freshly prepared 0.6 mL of 10 mM ice-cold NaBH_4_ solution was quickly injected into the mixed solution and magnetically stirred for 2 min. The mixture rapidly turned into a brownish-yellow color and was kept at room temperature for 2 h.

To prepare the growth solution, 5 mL of 0.01 M HAuCl_4_ and 0.35 mL of 10 mM AgNO_3_ were mixed with 95 mL of 0.1 M CTAB, followed by adding a freshly prepared 0.55 mL of 0.1 M AA solution to reduce the Au^3+^ to Au^0^. A seed solution of 120 μL was gently added into the resultant solution and was continuously stirred for 2 min, and then the reaction mixture was stored at 30°C for at least 3 h without agitation.

### Synthesis of AuNRs@SiO_2_ with different shell thickness

The silica-coated AuNRs were achieved by a modified StÖber method [24], and the silica shells with different thicknesses were obtained by adding varying amounts of TEOS. Briefly, 10 mL of the as-prepared AuNRs solution was centrifuged at 10,000 rpm for 30 min twice to remove the excess CTAB surfactant and was re-dispersed in 10 mL pure water. Subsequently, 100 μL of 0.1 M NaOH solution was added into the AuNRs solution under gentle stirring to adjust the pH value to 10.0. After that, 5, 8, 10, 15, 20, or 25 μL of TEOS/methanol (*V*/*V*: 1/4) solution together with 10 μL of APTMS/methanol (*V*/*V*: 1/49) solution was injected three times at a 30-min interval to form the silica shell. The reaction was allowed to be gently stirred for 24 h, and the resultant colloidal solution was centrifuged at 7,500 rpm for 30 min twice to remove the unreacted TEOS and APTMS.

### Covalent binding of AlC_4_Pc to AuNRs@SiO_2_

The covalent binding of AlC_4_Pc to the surface of amine-capped AuNRs@SiO_2_ was performed by using EDC and NHS cross-linking procedure. In a typical coupling reaction, 5 μL of 10 μM AlC_4_Pc was pre-activated by an EDC/NHS solution for 30 min and then added into 2 mL of 0.4 nM AuNRs@SiO_2_ solution. The mixed solution was incubated with gentle shaking at room temperature for 6 h. The unlinked AlC_4_Pc molecules were separated from AuNRs@SiO_2_ NPs after synthesis completion via centrifugation at 7,500 rpm for 30 min three times. By measuring the fluorescence intensity of every supernatant solution, the amount of unlinked AlC_4_Pc was deduced via a calibration curve plotted with known concentrations.

### Characterization

Transmission electron microscopy (TEM) images were acquired by using a JEM-2100 HRTEM (JEOL, Akishima-shi, Japan) operating with an accelerating voltage of 200 kV. The absorption spectra were measured using a UV-1750 UV-vis spectrophotometer (Shimadzu, Kyoto, Japan). The fluorescence emission spectra and fluorescence lifetime decay curves were obtained by FluoroMax-4 spectrofluorometer (HORIBA Jobin Yvon, Palaiseau, France); fluorescence lifetime decay curves were measured with a pulsed 591-nm nano LED as the excitation source. Detection of singlet oxygen generation was obtained using a FLS 920 spectrofluorimeter (FLS 920 spectrofluorimeter, Edinburgh, UK) with an excitation wavelength at 380 nm in the dark room.

### Fluorescence spectroscopic measurements

Two milliliter of AuNRs@SiO_2_-AlC_4_Pc with different shell thickness solution and free AlC_4_Pc solution were prepared for the fluorescence spectroscopic measure. The concentration of AlC_4_Pc in each sample was normalized by the fluorescence calibration curve. The fluorescence enhancement factor (EF_MEF_) was defined as Equation (1):
1

where *I*_*i*_ is the fluorescence intensity of AuNRs@SiO_2_-AlC_4_Pc varying with shell thickness, *I*_0_ is the fluorescence intensity of free AlC_4_Pc in the same amount, and *I*_background_ is the fluorescence intensity of pure water.

The fluorescence lifetime was measured with the method of time-correlated single photon counting (TCSPC). The TCSPC was based on the detection of single photons of a periodical light signal and the reconstruction of the waveform from the time measurements. The fluorescence intensity decays were analyzed in terms of the multi-exponential model (Equation 2):
2

where *α*_*i*_ is the amplitudes and *τ*_*i*_ is the decay times of each exponential component, respectively. The average lifetime was given by Equation (3):
3

With the numerous measurements, the average fluorescence lifetimes in different cases were determined.

### Measurement of singlet oxygen (^1^O_2_) generation

ABDA, a chemical-sensitive probe of ROS, was used to monitor the generation of ^1^O_2_[25]. In the photochemical experiment, 5 μL of 10 μM ABDA in DMSO solution was added to 2 mL of 0.4 nM AuNRs@SiO_2_-AlC_4_Pc with different shell thickness and free AlC_4_Pc solution, respectively. The mixtures were photo-activated by using an LED lamp excitation at 680 nm (10 mW cm^−2^), and the generation of ^1^O_2_ was measured by recording the fluctuation of ABDA fluorescence intensity after different amount of light exposures.

## Results and discussion

### Synthesis and characterization of AuNRs@SiO_2_-AlC_4_Pc

**Figure 1:**
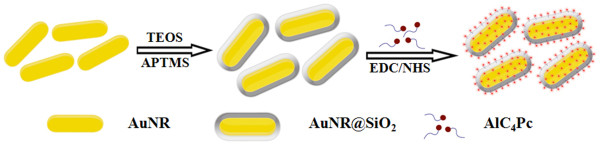
**Schematic description of the synthesis of AuNRs@SiO**_**2**_ **-AlC**_**4**_ **Pc.**

**Figure 2:**
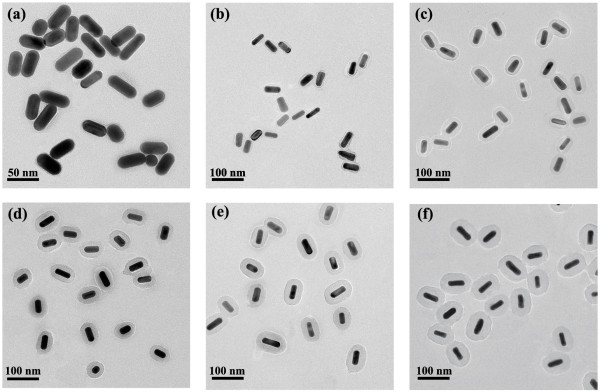
**TEM images of AuNRs@SiO**_**2**_ **-AlC**_**4**_ **Pc NPs with different shell thickness. (a)** 2.1, **(b)** 6.2, **(c)** 10.6, **(d)** 14.7, **(e)** 18.9, and **(f)** 28.2 nm.

The formation process of AuNRs@SiO_2_-AlC_4_Pc was depicted in Figure 1. The size and morphology of samples were shown in Figure 2. The average length and diameter of AuNRs core were measured to be 46.8 ± 3.2 and 19.4 ± 1.1 nm. By simply varying the amount of added TEOS, uniform silica spacer shells with desired the thickness of 2.1, 6.2, 10.6, 14.7, 18.9, and 28.2 nm were synthesized, respectively (Figure 2a,b,c,d,e,f). The easily tunable feature of these amorphous silica shells would serve them well as spacer layer between the AuNRs and AlC_4_Pc to get an optimum fluorescence enhancement effect.

### Spectral analysis of AuNRs@SiO_2_-AlC_4_Pc

**Figure 3:**
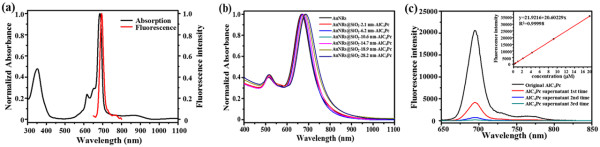
**Absorption and fluorescence spectra, normalized absorbance, and fluorescence spectra. (a)** Absorption and fluorescence spectra of AlC_4_Pc; **(b)** normalized absorbance of AuNRs@SiO_2_-AlC_4_Pc with different shell thickness; and **(c)** fluorescence spectra of original AlC_4_Pc and every supernatant. Inset: fluorescence standard curve of AlC_4_Pc over the concentration range 1 to 20 μM.

The AlC_4_Pc used in this study is one of the second generation photosensitizers that have good optical properties for near infrared fluorescence imaging [26]. The absorption and fluorescence emission spectra of AlC_4_Pc were presented in Figure 3a; AlC_4_Pc displayed two absorption bands located at 360 nm (B-band) and 685 nm (Q-band), and the fluorescence band was centered at 695 nm. This Q-band fitted well for PDT applications, as red light is commonly used in PDT for its better penetration into the tissue [27]. The absorption spectra of the obtained AuNRs showed two typical surface plasmon resonances (SPR) bands with the transverse one centered at 515 nm and the longitudinal one centered at 668 nm. Moreover, the longitudinal SPR band underwent an obvious red shift from 5 to 22 nm after coating the surface with silica shell (Figure 3b). This spectral displacement could be attributed to the increased in the effective refractive index of the medium around the AuNRs [24]. After successfully linking of AlC_4_Pc molecules, the sharp absorption peak of AlC_4_Pc has little influence on AuNRs because the amount of AlC_4_Pc linked on each nanoparticle was controlled as low as possible to avoid the self-quenching. To calculate the amount of unloaded AlC_4_Pc, the fluorescence intensities of the supernatants and the original AlC_4_Pc solution were analyzed by the standard curve (Figure 3c). It was indicated that the average number of AlC_4_Pc molecules linked to each nanoparticle was 38.

**Figure 4:**
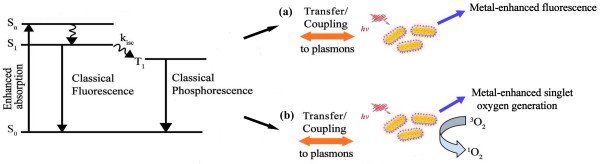
**Schematic Jablonski diagram for photosensitizer and mechanism of (a) metal-enhanced fluorescence and (b) singlet oxygen generation.**

Previous reports have proposed that a remarkable fluorescence enhancement was achieved when the emission band of fluorophores overlapped with the SPR band of MEF substrate [28, 29]. For the mechanism of MEF, it was commonly accepted that the excited fluorophore energy could be transferred to the surface plasmon modes of metal and then partly be re-radiated to the far-field or be quenched by a non-radiative decay pathway when the energy of the light-emitting fluorophore and the surface plasmon energy of metal were match well [14, 29, 30]. It was worthy to notice that the fluorescence band of AlC_4_Pc overlapped well with the longitudinal SPR band of AuNRs. The maximum spectra matching between them could result in an efficient energy transfer, which might strongly meet the fundamental requirement of MEF (Figure 4a).

### Distance-dependent MEF of AuNRs@SiO_2_-AlC_4_Pc

**Figure 5:**
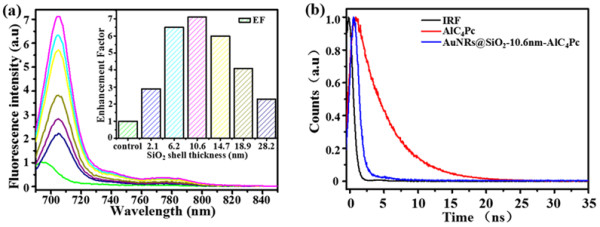
**Fluorescence spectra and fluorescence decay. (a)** Fluorescence spectra corresponding to the samples in Figure 1a,b,c,d,e,f. Inset: Distance-dependent fluorescence enhancement factor of AuNRs@SiO_2_-AlC_4_Pc. **(b)** Fluorescence decay of AuNRs@SiO_2_-AlC_4_Pc with 10.6 nm shell thickness and free AlC_4_Pc. The instrument response function (IRF) is included *λ*_em_ = 591 nm.

As depicted in Figure 5a, a 2.9-fold fluorescence enhancement was observed when the AuNRs-AlC_4_Pc separation distance was 2.1 nm. With an increase of the spacer thickness from 2.1 to 10.6 nm, the enhancement factor of MEF gradually increased and reached its maximum of 7.1 at 10.6 nm. This could be explained by that with the increase of silica shell, the local electromagnetic field enhancement effect became remarkable while the non-radiative decay from AlC_4_Pc to gold core could be negligible due to the short-range effect [9, 15]. However, a further increase of silica layer thickness caused an obvious decline of EF_MEF_. Because the positions of AlC_4_Pc molecules were beyond the effective electromagnetic field enhancement range, thus the interaction of AlC_4_Pc with gold core were weakened and gradually disappeared. Therefore, when the AlC_4_Pc molecule was too close or too far to the AuNRs, MEF effect could be diminished by non-radiative energy transfer or weak electromagnetic field, respectively.

To further confirm whether the observed fluorescence enhancement was attributed to the resonance interaction, a fluorescence lifetime measurement was carried out. As can be observed in the Figure 5b, the fluorescence intensity decays were fitted in terms of the multi-exponential model. The fluorescence lifetime of AlC_4_Pc was dramatically shortened from 4.78 to 0.515 ns after linking on the AuNRs@SiO_2_, when the distance between AlC_4_Pc and AuNRs was 10.6 nm.

It is well-known that when a fluorophore radiates into a homogeneous medium, the quantum yield (*Q*_0_) and lifetime (τ_0_) can be given by Equations (4) and (5) [31, 32]:
45

where *Г* is the fluorophore radiative rate and *k*_nr_ is the non-radiative rates of the fluorophore. For a fluorophore localized near the metal particle, both the radiative and the non-radiative decay rates are modified by the metal substrate and given an additional *Γ*_*m*_ to the radiative rate and *k*_m_ to the non-radiative rate, as shown in Equations (6) and (7) [33]:
67

Comparing with the free-space radiation, the increase in the radiative rate would directly increase the quantum yield and decrease the lifetime of a fluorophore. The increased radiative rate of fluorophore, together with the concentrated local electromagnetic field of metal substrate, can give a well explanation for the MEF phenomenon of AuNRs@SiO_2_-AlC_4_Pc [34].

### Effects of different shell thickness on singlet oxygen generation

**Figure 6:**
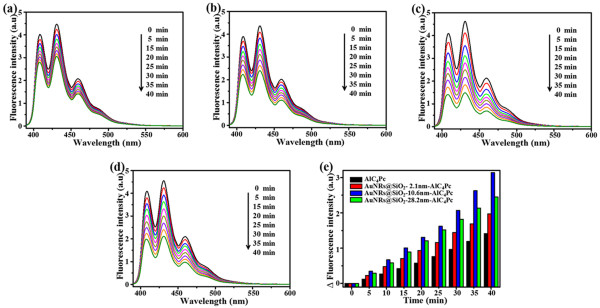
**Photobleaching of ABDA.** Photobleaching of ABDA by ^1^O_2_ generated by **(a)** free AlC_4_Pc as control; **(b)**-**(d)** AuNRs@SiO_2_-AlC_4_Pc with 2.1, 10.6, and 28.2 nm shell thickness; and **(e)** time-dependent decrease in ABDA fluorescence (Δ*F*) as a function of irradiation time, corresponding to **(a)**-**(d)**.

Early studies have indicated that both of the fluorescence and the ^1^O_2_ generation would be effectively inhibited by non-radiative decay from the excited PSs to metal when the PSs were close proximity to the metal surface [35]. In our study, three groups of AuNRs@SiO_2_-AlC_4_Pc NPs with 2.1, 10.6, and 28.2 nm silica shell were incubated with ABDA to assess the influence of shell thickness on the ^1^O_2_ generation. After being exposed in different amount of light exposure, the fluorescence intensity of ABDA was decreased because of the formation of its endoperoxide in the presence of ^1^O_2_ (Figure 6a,b,c,d). The decreased amount of ABDA fluorescence at 431 nm can be used to estimate the relative yield of ^1^O_2_ produced from AlC_4_Pc. The time-related fluorescence decline of ABDA in different AuNRs@SiO_2_-AlC_4_Pc solutions and free AlC_4_Pc samples were directly compared in Figure 6e. Among the tested samples, AuNRs@SiO_2_-AlC_4_Pc with 10.6 nm silica shell exhibited the highest photo-oxidation efficiency with 2.1-fold higher than that of the free AlC_4_Pc solution. The accelerated photobleaching of ABDA was mainly affected by the AlC_4_Pc-AuNRs interaction. As mentioned above, the concentrated electromagnetic field may play a key role in the enhancement of ^1^O_2_ generation. ^1^O_2_ generation could also be enhanced by the promotion of intersystem crossing and the yield of triplet state, which probably result from the increased population of singlet excited state of AlC_4_Pc [21, 36] (Figure 4b). Furthermore, a shorter lifetime leads to a less possibility of photobleaching, and then more excitation-emission cycles occur prior to photobleaching of PSs during the excited state [10]. The amplified production of ^1^O_2_ and great enhancement of fluorescence intensity of AuNRs@SiO_2_-AlC_4_Pc could serve them well as a theranostic agent for fluorescence imaging-guided cancer treatment.

## Conclusions

In summary, the AuNRs@SiO_2_ core-shell NPs linked with AlC_4_Pc were prepared to study the MEF phenomenon and ^1^O_2_ generation. As we have shown, the fluorescence of AlC_4_Pc was significantly enhanced by optimizing the distance between AlC_4_Pc and AuNRs. Meanwhile, ^1^O_2_ generation could also be increased probably by the promotion of intersystem crossing and triplet state yield. Co-enhancement of fluorescence intensity and ^1^O_2_ generation reached their maximum effect when the distance between AlC_4_Pc and AuNRs was 10.6 nm. Although the mechanism of MEF and metal-enhanced ^1^O_2_ generation are still under investigation, these results provide a desirable insight towards evaluating and improving the performance of AuNRs-based NPs in fluorescence imaging and PDT.

## References

[1] Anker JN, Hall WP, Lyandres O, Shah NC, Zhao J, Van Duyne RP (2008). Biosensing with plasmonic nanosensors. Nat Mater.

[2] Mayer KM, Lee S, Liao H, Rostro BC, Fuentes A, Scully PT, Nehl CL, Hafner JH (2008). A label-free immunoassay based upon localized surface plasmon resonance of gold nanorods. Acs Nano.

[3] Haes AJ, Van Duyne RP (2002). A nanoscale optical biosensor: sensitivity and selectivity of an approach based on the localized surface plasmon resonance spectroscopy of triangular silver nanoparticles. J Am Chem Soc.

[4] Raschke G, Kowarik S, Franzl T, Sönnichsen C, Klar T, Feldmann J, Nichtl A, Kürzinger K (2003). Biomolecular recognition based on single gold nanoparticle light scattering. Nano Lett.

[5] Nie S, Emory SR (1997). Probing single molecules and single nanoparticles by surface-enhanced Raman scattering. Science.

[6] Qian X, Peng X-H, Ansari DO, Yin-Goen Q, Chen GZ, Shin DM, Yang L, Young AN, Wang MD, Nie S (2007). In vivo tumor targeting and spectroscopic detection with surface-enhanced Raman nanoparticle tags. Nat Biotechnol.

[7] Durr NJ, Larson T, Smith DK, Korgel BA, Sokolov K, Ben-Yakar A (2007). Two-photon luminescence imaging of cancer cells using molecularly targeted gold nanorods. Nano Lett.

[8] El-Sayed IH, Huang XH, El-Sayed MA (2005). Surface plasmon resonance scattering and absorption of anti-EGFR antibody conjugated gold nanoparticles in cancer diagnostics: applications in oral cancer. Nano Lett.

[9] Anger P, Bharadwaj P, Novotny L (2006). Enhancement and quenching of single-molecule fluorescence. Phys Rev Lett.

[10] Zhang J, Fu Y, Chowdhury MH, Lakowicz JR (2007). Metal-enhanced single-molecule fluorescence on silver particle monomer and dimer: coupling effect between metal particles. Nano Lett.

[11] Amiot CL, Xu S, Liang S, Pan L, Zhao JX (2008). Near-infrared fluorescent materials for sensing of biological targets. Sensors-Basel.

[12] Lakowicz JR, Shen Y, D'Auria S, Malicka J, Fang J, Gryczynski Z, Gryczynski I (2002). Radiative decay engineering: 2. Effects of silver island films on fluorescence intensity, lifetimes, and resonance energy transfer. Anal Biochem.

[13] Fu Y, Lakowicz JR (2006). Enhanced fluorescence of Cy5-labeled DNA tethered to silver island films: fluorescence images and time-resolved studies using single-molecule spectroscopy. Anal Chem.

[14] Aslan K, Leonenko Z, Lakowicz JR, Geddes CD (2005). Annealed silver-island films for applications in metal-enhanced fluorescence: interpretation in terms of radiating plasmons. J Fluoresce.

[15] Chhabra R, Sharma J, Wang H, Zou S, Lin S, Yan H, Lindsay S, Liu Y (2009). Distance-dependent interactions between gold nanoparticles and fluorescent molecules with DNA as tunable spacers. Nanotechnology.

[16] Zhou Z, Huang H, Chen Y, Liu F, Huang CZ, Li N (2014). A distance-dependent metal-enhanced fluorescence sensing platform based on molecular beacon design. Biosens Bioelectron.

[17] Jin Y, Gao X (2009). Plasmonic fluorescent quantum dots. Nat. Nanotechnol.

[18] Zhang J, Ma N, Tang F, Cui Q, He F, Li L (2012). pH-and glucose-responsive core-shell hybrid nanoparticles with controllable metal-enhanced fluorescence effects. ACS Appl Mater Inter.

[19] Tang F, Ma N, Wang X, He F, Li L (2011). Hybrid conjugated polymer-Ag@ PNIPAM fluorescent nanoparticles with metal-enhanced fluorescence. J Mater Chem.

[20] Tovmachenko OG, Graf C, van den Heuvel DJ, van Blaaderen A, Gerritsen HC (2006). Fluorescence enhancement by metal-core/silica-shell nanoparticles. Adv Mater.

[21] Zhang Y, Aslan K, Previte MJ, Geddes CD (2008). Plasmonic engineering of singlet oxygen generation. Proc Natl Acad Sci U S A.

[22] Chen F, Xu D (1990). Synthesis of water soluble phthalocyanines. Chin J Org Chem.

[23] Nikoobakht B, El-Sayed MA (2003). Preparation and growth mechanism of gold nanorods (NRs) using seed-mediated growth method. Chem Mater.

[24] Wu C, Xu Q-H (2009). Stable and functionable mesoporous silica-coated gold nanorods as sensitive localized surface plasmon resonance (LSPR) nanosensors. Langmuir.

[25] Qian HS, Guo HC, Ho PCL, Mahendran R, Zhang Y (2009). Mesoporous-silica-coated up-conversion fluorescent nanoparticles for photodynamic therapy. Small.

[26] Wang F, Chen X, Zhao Z, Tang S, Huang X, Lin C, Cai C, Zheng N (2011). Synthesis of magnetic, fluorescent and mesoporous core-shell-structured nanoparticles for imaging, targeting and photodynamic therapy. J Mater Chem.

[27] Fomina N, Sankaranarayanan J, Almutairi A (2012). Photochemical mechanisms of light-triggered release from nanocarriers. Adv Drug Deliv Rev.

[28] Chen Y, Munechika K, Ginger DS (2007). Dependence of fluorescence intensity on the spectral overlap between fluorophores and plasmon resonant single silver nanoparticles. Nano Lett.

[29] Tam F, Goodrich GP, Johnson BR, Halas NJ (2007). Plasmonic enhancement of molecular fluorescence. Nano Lett.

[30] Aslan K, Malyn SN, Geddes CD (2008). Angular-dependent metal-enhanced fluorescence from silver island films. Chem Phys Lett.

[31] Lakowicz JR (2005). Radiative decay engineering 5: metal-enhanced fluorescence and plasmon emission. Anal Biochem.

[32] Lakowicz JR, Masters BR (2008). Principles of fluorescence spectroscopy. J Biomed. Opt.

[33] Lakowicz JR (2001). Radiative decay engineering: biophysical and biomedical applications. Anal Biochem.

[34] Zhang J, Fu Y, Chowdhury MH, Lakowicz JR (2008). Plasmon-coupled fluorescence probes: effect of emission wavelength on fluorophore-labeled silver particles. J Phys Chem C.

[35] Cheng YC, Samia A, Meyers JD, Panagopoulos I, Fei B, Burda C (2008). Highly efficient drug delivery with gold nanoparticle vectors for in vivo photodynamic therapy of cancer. J Am Chem Soc.

[36] Zhang Y, Aslan K, Previte MJ, Geddes CD (2007). Metal-enhanced singlet oxygen generation: a consequence of plasmon enhanced triplet yields. J Fluoresc.

